# Encapsulation of *Polygonum bistorta* root phenolic compounds as a novel phytobiotic and its protective effects in the mouse model of enteropathogenic *Escherichia coli* infection

**DOI:** 10.1186/s12906-023-03868-2

**Published:** 2023-02-15

**Authors:** Zahra kadkhoda Mezerji, Reza Boshrouyeh, Seyedehfarnaz Hafezian Razavi, Shaghayegh Ghajari, Hasti Hajiha, Negin Shafaei, Ehsan Karimi, Ehsan Oskoueian

**Affiliations:** 1 Department of Biology, Mashhad Branch, Islamic Azad University, Mashhad, Iran; 2 Department of Research and Development, Arka Industrial Cluster, Mashhad, Iran

**Keywords:** Phytogenic, Delivery, Antibacterial agent, Natural antibiotic, Microencapsulation, Antibiotic-alternative

## Abstract

**Background:**

Microencapsulation technology is the fundamental delivery system for encapsulating the natural bioactive compounds especially phenolic in order to developing bioavailability, stability and controlling release. This study was conducted to determine the antibacterial and health-promoting potential of the phenolic rich extract (PRE)-loaded microcapsules obtained from *Polygonum bistorta* root as a dietary phytobiotic in mice challenged by enteropathogenic *Escherichia coli* (*E. coli*).

**Method:**

The PRE was obtained from *Polygonum bistorta* root using fractionation by different polarity solvents and the highest PRE was encapsulated by the combination of modified starch, maltodextrin, and whey protein concentrate as wall materials using a spray dryer. Then, the physicochemical characterization (particle size, zeta potential, Morphology and polydispersity index) of microcapsules have been assessed. For the invivo study, 30 mice at five treatment were designed and antibacterial properties were analyzed. Furthermore, relative fold changes in the ileum population of *E. coli* was investigated using Real time PCR.

**Results:**

The encapsulation of PRE resulted in the production of phenolic enriched extract-loaded microcapsules (PRE-LM) with a mean diameter of 330 nm and relatively high entrapment efficiency (87.2% w/v). The dietary supplementation of PRE-LM improved weight gain, liver enzymes, gene expression, morphometric characteristics of the ileum and decreased the population of *E. coli* present in the ileum significantly (*p* < 0.05).

**Conclusion:**

Our funding suggested PRE-LM as a promising phytobiotic against *E. coli* infection in mice.

## Background

Nowadays polyphenols have been applied as a natural phytobiotic and antioxidant supplement to fight enteropathogens and improve gut health and function [1] Phenolic compounds are universally distributed in plants and phenolics such as gallic acid, syringic acid, ferulic acid, catechin, ellagic acid, naringin, and chrysin are known for their antimicrobial and antioxidant activities [2]. Phenolic compounds' biological activity are also greatly affected by their interactions with other biomolecules. For instance, the solubility and bioactivity of tannins may also be altered by the presence of various molecules, including polysaccharides and proteins. The strong interactions with other biomolecules are likely to interfere with the bioavailability and biological activities of phenolic compounds [3].

*Polygonum bistorta* L. (*Polygonacea* family) is known as Bistort or Snakeroot, which is perennial herbaceous flowering with a slender stem distributed in Europe, Asia, North America, and India. It is a popular medicinal plant in many traditional medicines for its medicinal properties such as controlling bleeding, astringent, diuretic, and expectorant. Among the different aerial parts, the root has been widely used for treating dysentery, sepsis, exogenous heat, cough, and microbial infections [4, 5]. The former investigation has confirmed the main bioactive compound constituents in this plant [5, 6]. A study was performed by Pirvu et al. [7] indicated the presence of caffeic acid, gallic acid [8], ellagic acid, and ferulic acid [9] as the main phenolic compounds, and rutin [10], hyperine, quercitrin, isoquercitrin [11], miquelianin [9] as the important flavonol glycosides identified in this herbal medicine [7]. These natural compounds are known for their antimicrobial activities. Similar research elucidated the appreciable antimicrobial potential of various parts of this plant against pathogenic bacteria such as *Paenibacillus larvae*, *Melissococcus plutonius* and *Bacillus subtilis* [12]. A recent experiment has been done by Pawłowska et al. [13] indicated that the Flavon-3-ols and galloylglucose derivatives as natural products possess antimicrobial activity against skin pathogens [13]. While the various type of bioactive compounds especially phenolic compounds have been found in the root of *Polygonum bistorta* L. therefore, it could be an interesting source for natural phytochemicals with health-promoting properties in the medicinal, cosmeceuticals, nutraceuticals, and pharmaceutical industries. Despite the various biological activities of the phenolic compounds for the treatment of diseases, their less bioavailability, low absorption, and sensitivity to oxidation are the main problems of these compounds [14, 15]. Therefore, the encapsulation of these phytochemicals is suggested to overcome these limitations facilitate their release, and enhance their bioavailability [16].

The encapsulation technology has great attention and interest in the delivery of bioactive compounds as this technology could help in maintaining all features of bioactive compounds such as potent and strong protection. This technology can be categorized into three parts: physical methods like spray drying, a chemical procedure like molecular inclusion, and a physicochemical process such as liposome encapsulation [17–19]. Among all described techniques spray drying is widely performed for encapsulation of bioactive compounds. In this technology, different types of carriers are commonly used including starch, maltodextrin, gum, and whey protein concentrate. In the present study, we attempted to encapsulate the phenolic-rich extract obtained from *Polygonum bistorta* L. root and to determine its antioxidant, and antibacterial activities in the mouse model of enteropathogenic *Escherichia coli* infection.

## Materials and method

### Materials

The dry roots of *Polygonum bistorta* were purchased from the herbal market of Shirvan, North Khorasan, IRAN. The voucher spiceman was deposited in islamic azad university herbarium by 10,536 IAUM code number. For encapsulation, the wall material such as modified starch (MS) (HI-CAP® 100, Ingredion, Humberg, Germany) and maltodextrin (MD) (DE = 18–20; Foodchem, China) and whey protein concentrate 80% (w/w) (WPC) (Hilmar, CA, USA) was purchased. The foodborne pathogen *Escherichia coli* (*E. coli* O157: H7) was purchased from the microbial culture collection of the Razi Vaccine and Serum Research Institute, Karaj, Iran. The RNeasy mini kit, QIAamp DNA Stool Mini Kit was obtained from Qiagen Germany. The cDNA synthesis kit and SYBER green were bought from Biofact, Korea. All the other solvents and chemicals not mentioned here were purchased from Merck, Germany.

### Obtaining phenolic-enriched extract

The root part of the plant was ground to the fine powder using laboratory grinder. For obtaining of phenolic-enriched extract, 100 g of dried root powder was added into the 2L round bottom flask and 1000 mL of methanol (80% v/v), and 50 mL of hydrochloric acid (6 M) were added. The mixture was refluxed (90 °C, 2 h) [20]. The obtained extract was filtered and the solvent was evaporated (60 °C) by using a rotary evaporator (Buchi, Flawil, Switzerland) [21]. To obtain the phenolic-enriched extract (PRE) the dried extract was re-extracted with different polarity solvents including hexane, chloroform, ethyl acetate, n-butanol, and water, respectively. For extract partitioning, 250 mL from each solvent was used and re-extraction was performed twice in a separating funnel. Finally, the obtained extract was filtered and concentrated using a rotary evaporator (Buchi. Switzerland). The total phenolic content of each extract was determined colorimetrically using a visible spectrophotometer (Novaspec II Visiblespectro, Japan) at 765 by Folin-ciocalteu assay [21], and results were reported as mg gallic acid equivalent (GA eq.) /g dry crude extract. The extract with the highest concentration of phenolic compounds is called phenolic-enriched extract (PRE) and is used for further experiments.

### Encapsulation process

A pilot plant scale spray dryer (Buchi, B-191, Switzerland) with counter-current airflow was utilized to encapsulate the PRE obtained from *P. bistorta* root [22]. The maltodextrin (70% w/w), modified starch (20% w/w), and whey protein concentrate (10% w/w) was used as wall materials according to our early studies [23–25]. Briefly, the PRE (8 g), tween 80 (3 g), and the 32 g of wall material with the respective ratio of 1:4 were added to 100 ml of water and mixed using a stirrer at 4 °C overnight for complete rehydration. The solution was homogenized using Ultra Turrax homogenizer (IKA, Germany) before spry drying at 10,000 rpm for 5 min. The temperature of the spray dryer was fixed between the range of 130 °C (inlet temperature) and 80 °C (outlet temperature), the pressure was 0.4 kg/cm^2^ and the flow rate was adjusted to 8 mL/min. The dried powder obtained is called PRE-loaded microcapsules (PRE-LM) and it was used for further investigations.

### Characterization of microcapsules

The particle size, zeta potential, and polydispersity index (PDI) of the capsules containing PRE of *P. bistorta* root were tested by Zetasizer (Malvern Instruments Ltd., UK). The morphology evaluation of microcapsules was carried out by Scanning Electron Microscopy (SEM). The total phenolic compounds in the capsules were determined using the Folin–Ciocalteau assay [21].

### Entrapment efficiency

The entrapment efficiency of PRE-LM was determined as described earlier with slight modifications [26, 27]. The dried microcapsules (100 mg) were dispersed in 1.5 ml of 80% (v/v) methanol and centrifuged (12,000 rpm, 5 min). The total phenolic compounds were determined in the supernatant by Folin–Ciocalteau assay. The pellet (precipitant) was re-extracted using 1.5 ml of 80% (v/v) methanol using a sonicator 60 kHz for the 30 s. The total phenolic compounds were determined in the extract obtained from the pellet. The experiment was repeated three times. Finally, the entrapment efficiency (EE) was calculated using the below formula.

$$\mathrm{EE} \left(\%\right)=100\times \frac{\mathrm{total phenolics in the pellet}-\mathrm{total phenolic in the supernatant}}{\mathrm{total phenolic in the pellet}}$$

### HPLC analysis of phenolic compounds

The profiling of natural phenolic compounds present in the PRE-LM was determined using high-performance liquid chromatography (HPLC) with a UV–Vis photodiode array (DAD) detector (Agilent-1200 series). The phenolic compounds present in the PRE-LM were extracted using 80% (v/v) methanol applying sonication for the complete extraction of phenolic compounds from microcapsules. The extract was used for HPLC analysis. The C18 analytical column (Intersil ODS-3 5um 4.6 × 150 mm Gl Science Inc. USA) was applied for identification of phenolic compounds. It equilibrated with 85% of deionized water as solvent A and 15% of acetonitrile as solvent B. Then the ratio of solvent B was enhanced to 85% in 50 min followed by decreasing solvent B to 15% in 55 min. This ratio was kept to 50 min for the next analysis. Gallic acid, syringic acid, vanillic acid, salicylic acid, caffeic acid, pyrogallol, catechin, cinnamic acid, ellagic acid, naringin, chrysin, and ferulic acid were the bioactive phenolic standards used in this research [28].

### Antioxidant and antibacterial activities

The antioxidant potential of the PRE-LM was evaluated using DPPH radical scavenging activity. Vitamin C was used as antioxidant standard [29]. The antibacterial properties of PRE-LM were determined against *E. coli* (O157: H7) and data was reported as Minimum Inhibitory Concentration (MIC) using microdilution method and the positive control was oxytetracycline [30]. All the experiments mentioned here were run in triplicates.

### Animal trial

The 30 Balb/c male mice with a respective weight range of 28–30 g were provided by the Razi Vaccine and Serum Research Institute of Mashhad, Mashhad, Iran. To acclimatize the animal, they were kept in cages for 10 days (12 h light /dark cycle) and received food and water. After that, they were randomly divided into five groups as described in Table 1. All mice received experimental treatments for 4 weeks and the oral infection was performed through a gavage needle using 10^8^ CFU of *E. coli* once on day 21. Animals were monitored daily for general health and the amount of food eaten. At the end of the experiment (Day 28), the mice were euthanized with pentobarbital-HCL (50 mg/kg, i.p.) and sacrificed. The blood, liver, and ileum samples were collected immediately and used for liver enzyme analysis, lipid peroxidation assay, gene expression analysis, and morphometric evaluation of the ileum.

**Table 1:** Experimental treatments

Treatments	Diets	Infection	Sampling
T1	Normal diet	**-**	Day 28
T2	Normal diet	Infected on Day 21 through oral gavage (10^8^ CFU of *E. coli* O157: H7)	
T3	A normal diet supplemented with microcapsules (150 mg total phenolic compounds^a^ (GA eq.) /Kg BW)		
T4	A normal diet supplemented with microcapsules (300 mg total phenolic compoundsa^a^ (GA eq.) /Kg BW)		
T5	Normal diet + 5 mg/kg of oxytetracycline as reference antibiotic/Kg BW		

^a^The total phenolic compounds in the microcapsules were determined using folin-ciocalteu method as described in the methods section

### Liver enzymes and lipid peroxidation assay

The biochemical assay of serum for liver enzymes including alkaline phosphatase (ALP), alanine aminotransferase (ALT), and aspartate aminotransferase (AST) was carried out on an automated chemistry analyzer (Roche, Hitachi 902 analyzer, Japan). The lipid peroxidation of the liver for each treatment was evaluated using the determination of malondialdehyde (MDA) content in the liver tissue lysate by thiobarbituric acid reactive substances as described earlier [31].

### Gene expression analysis

The ileum tissues were separated and kept in liquid nitrogen at the end of in vivo study. Then, the RNA was extracted using the RNeasy Mini kit (Qiagen, Hilden, Germany) and immediately proceed to cDNA synthesis using a cDNA synthesis kit (Biofact Korea). The real-time PCR program was adjusted at 95 °C for 5 min (1X), 95 °C for 25 s, then 58 °C for the 30 s, and 72 °C for 30 s (35X). Finally, the expressions of claudin, occludin, and mucin as major biomarker genes in nutrient absorption and defense system in the ileum were determined. The expressions of genes were normalized to β-actin as a reference gene [32]. The list of primers used in the current study are Claudin-1 (F: tccttgctgaatctgaaca; R: agccatccacatcttctg); Claudin-2 (F: gtcatcgcccatcagaagat; R: actgttggacagggaaccag); Occludin (F: actcctccaatggacaagtg; R: ccccacctgtcgtgtagtct); Mucin-2 (F: gatggcacctacctcgttgt; R: gtcctggcacttgttggaat) and Beta-actin (F: gctgagagggaaatcgtgcgtg; R: ccagggaggaagaggatgcgg) [33, 34].

### Quantification of E. coli in the ileum digesta

The major sites of microbial fermentation, propagation, and colonization of enteropathogens in the monogastric are the ileum, which is why in this study the population of *E. coli* was analyzed only in the ileum section. The entire ileum digesta was taken and mixed and 100 mg of the digesta was used for the DNA extraction using QIAamp DNA Stool Mini Kit (Germany). The quantitative real-time PCR was applied using the SYBR Green PCR Master Mix (Biofact, Korea). The fold changes for the *E. coli* in the ileum digesta was determined using the 2^−∆∆Ct^ method as described earlier [35, 36]. The primer used in this study are study are *E. coli* (O157:H7) ( F: ttaccagcgataccaagagc; R: caacatgaccgatgacaagg) [37] and Total bacteria (F: cggcaacgagcgcaaccc; R: ccattgtagcacg tgtgtagcc) [38].

### Statistical analysis

The ANOVA test, using the GLM procedure of SAS (Ver. 9.1) was considered to analyze the obtained results. The significance was confirmed by Duncan's multiple range test. The *p-*value of < 0.05 was used as the criterion for a statistically significant difference. All experiments were done in triplicate and the results are presented as mean values ± standard deviation (mean ± SD) or mean values + standard error of the means (SEM).

## Results and discussion

### Extract partitioning

The application of solvents with different polarities for the partitioning of phenolic compounds from *P. bistorta* root produced different partitions with various amounts of phenolic compounds. The highest amount of phenolic content among partitions was observed in ethyl acetate extract with respective values of 256.7 ± 3.67 mg/g GAE followed by n-butanol (61.2 ± 5.03) > water (34.1 ± 4.27) > chloroform (21.7 ± 3.63) > hexane (18.7 ± 4.82) mg/g dried extract, respectively. Based on these results, the ethyl acetate extract was used for further evaluations as a phenolic enriched extract (PRE). These results indicated that the polarity of solvents could influence the extraction of phenolic compounds. The earlier publications reported the phenolic compounds as moderately polar compounds hence they tended to accumulate in the medium polar solvent such as ethyl acetate. The results obtained in this experiment were similar to other reports which observed the highest phenolic content in the ethyl acetate extract among other solvents such as hexane, chloroform, n-butanol, and water [39–41]. A similar observation was made by Abdelwahab et al. [42] and Kaur et al. [43] who observed the highest phenolic rich extract in the ethyl acetate extract when various polarity solvents were used for the extraction of *Orthosiphon stamineus* and *Chukrasia tabularis* respectively.

### Characteristics of PRE-LM

**Fig. 1:**
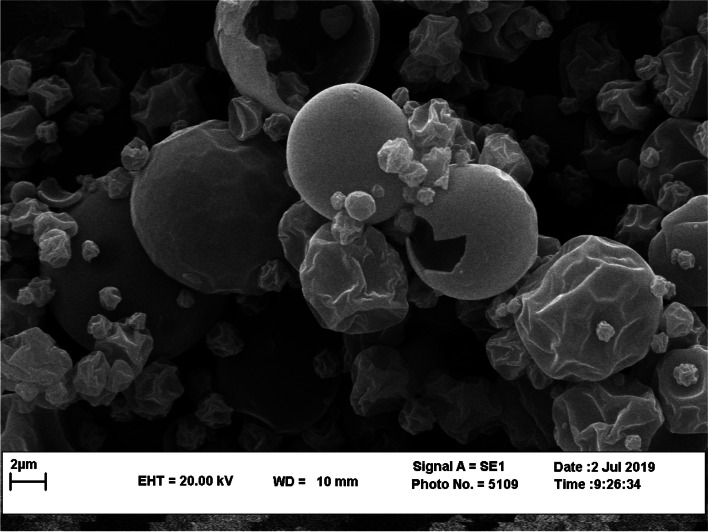
Scanning electron micrographs of microcapsules containing phenolic enriched extract-loaded microcapsules (PRE-LM) obtained from *P*. *bistorta* root

Table 2 illustrated the physicochemical traits of PRE-LM of *P*. *bistorta* roots. The encapsulation efficiency was ~ 87.2% (w/w) and surface phenolic compounds were 7.1% (w/w). The results indicated that the spray-dried technique could produce microcapsules with 330.3 nm in size with an acceptable PDI range (0.21) indicating the homogenous dispersion [44]. The total phenolic content of capsules was 172.6 ± 9.24 mg GA eq./g dry microcapsules. Furthermore, the SEM is applied to determine the surface morphology of materials and it is known as a valuable method for monitoring the size and formation of inclusion complexes. The SEM photomicrographs revealed that microcapsules are spherical and mostly with uniform and smooth cover with minimum fractures and signs of collapse (Fig. 1). The size of microcapsules in the SEM micrograph was consistent with the results obtained by Zetasizer. The encapsulation of plant bioactive compounds could protect the phenolics from oxidation, evaporation, and facilitate precision dosing. It is a low-cost and popular method for microencapsulation of bioactive compounds in the industry [45]. Consistent with the results obtained in this study, the early studies reported the encapsulation efficiency ranged from ~ 65 up to 95% (w/w) with the particle size ranging from 100 nm up to 4 µm. The early reports revealed the successful microencapsulation of polyphenols extracted from olive leaves, citrus processing by-products, grape pomace, and pomegranate peel and indicated that the encapsulation of phenolic compounds enhanced the solubility, functionality and shelf life of bioactive phenolic compounds [45–48].

**Table 2:** Physical and chemical characteristics of phenolic enriched extract-loaded microcapsules (PRE-LM) obtained from *P*. *bistorta* root^a^

Encapsulation efficiency (%)	Surface phenolics (%)	Particle size (nm)	Polydispersity index	Total phenolic content^b^ (mg GA eq./g dry microcapsules)
87.2 ± 6.33	7.1 ± 1.26	330.3 ± 6.89	0.21 ± 0.08	172.6 ± 9.24

^a^The analysis was performed in triplicates (*n* = 3)

^b^ mg gallic acid equivalent /g dry microcapsules

### HPLC analysis of PRE-LM

**Fig. 2:**
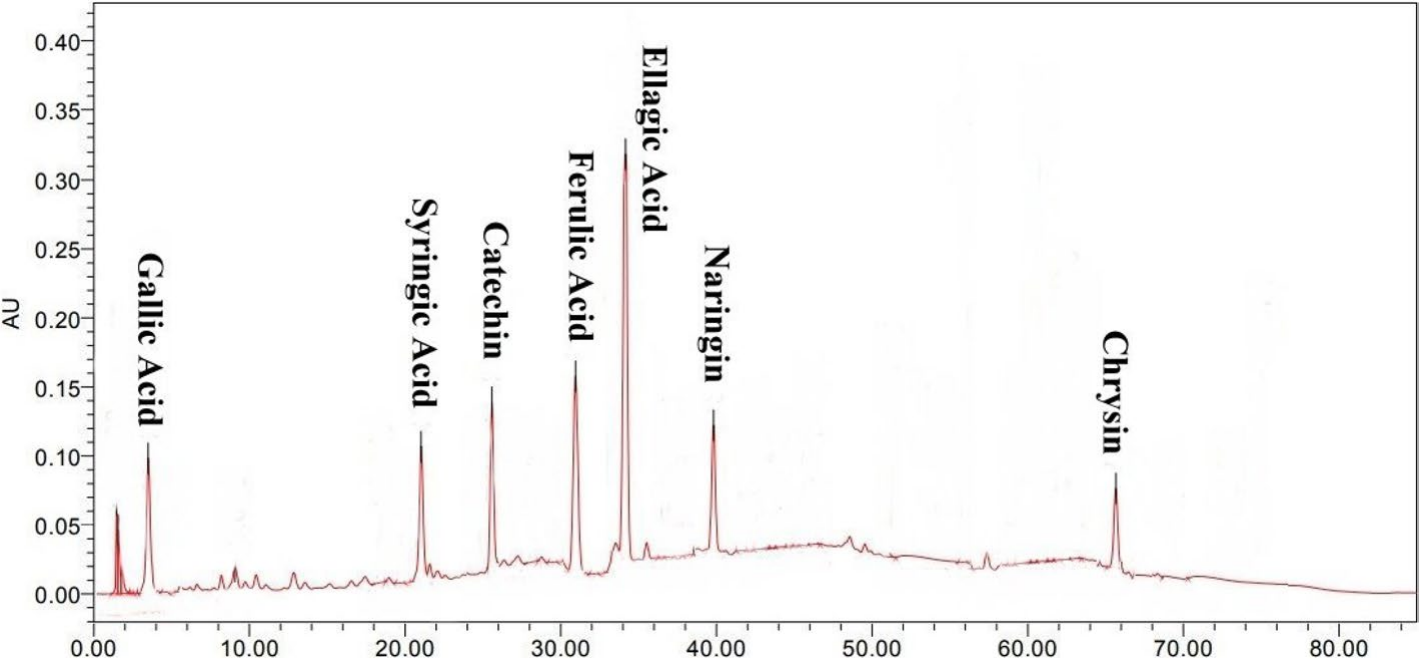
The RP-HPLC chromatogram of various phenolic compounds detected in phenolic enriched extract-loaded microcapsules (PRE-LM) obtained from *P*. *bistorta* root*.* The analysis was performed in triplicates (*n *= 3)

The HPLC profiling of PRE-LM from *P*. *bistorta* root indicated various phenolic compounds including gallic acid, syringic acid, ferulic acid, catechin, ellagic acid, naringin, and chrysin with the concentration ranging from 195 to 911 µg/g DW (Table 3). The HPLC chromatogram in Fig. 2 shows the various phenolic compounds in PRE-LM of *P*. *bistorta.*

**Table 3:** Phenolic compounds profile of the phenolic enriched extract-loaded microcapsules (PRE-LM) obtained from *P*. *bistorta* root

Phenolic compounds (µg/g DW)			
**Gallic Acid**	**Syringic Acid**	**Ferulic Acid**	**Catechin**
285.7 ± 1.6	392.4 ± 5.2	527.3 ± 4.1	208.6 ± 2.3
**Ellagic Acid**	**Naringin**	**Chrysin**	**-**
911.3 ± 6.8	315.7 ± 2.5	195.9 ± 2.7	-

The analysis was performed in triplicates (*n* = 3)

### Biological activities of PRE-loaded microcapsules

The antioxidant and antibacterial properties of PRE-LM have been shown in Table 4. The microcapsules manifest appreciable antioxidant by preventing the DPPH free radicals with IC_50_ values of 38.1 ± 4.18 µg/ml but the activities were lower than Vitamin C (17.1 ± 2.37 µg/ml) as the reference antioxidant. Besides that, the antibacterial potential of PRE-LM against the *E. coli* indicated that MIC values for the microcapsules and oxytetracycline as a reference antibiotic were 52.7 ± 2.86 and 19.2 ± 3.06 µg/ml, respectively. The moderate antibacterial and antioxidant activity of PRE-LM is probably attributed to the presence of phenolic compounds including gallic acid, syringic acid, ferulic acid, catechin, ellagic acid, naringin, and chrysin. It is difficult to say which phenolic compound mainly contributed in the antibacterial activity but most probably the synergistic effects of phenolic compounds may have resulted in the observed antibacterial activity. The mechanisms of action of phenolic compounds on bacterial cell have been partially attributed to inhibition of virulence factors such as enzymes and toxins, damage to the bacterial membrane, and suppression of bacterial biofilm formation [49]. The early studies reported the significant roles of these phenolic compounds in the antibacterial and antioxidant activity of plant extract as there was a direct relationship between the phenolic compounds with biological activities of plant extracts [50, 51].

**Table 4:** Antioxidant and antibacterial potential of phenolic enriched extract-loaded microcapsules (PRE-LM) obtained from *P*. *bistorta* root

Samples	DPPH scavenging activity (IC_50_)	MIC^a^ (µg/ml)
PRF-LM	38.1 ± 4.18	52.7 ± 2.86
Vitamin C	17.1 ± 2.37	-
Oxytetracycline	-	19.2 ± 3.06

The analysis was performed in triplicates (*n* = 3)

^a^minimum inhibitory concentration against *E. coli* O157: H7

### Animal trial

Table 5 shows the average daily weight gain and feed intake of mice receiving different treatments during the 28 days of study. It can be inferred from the results that the mice receiving normal food (T1) possessed the average daily weight gain with the value of 155.9 mg. In contrast, the mice challenged with enteropathogenic *E. coli* showed the lowest average daily weight gain (126.3 mg). It was interesting that the mice that received 150 and 300 mg phenolic compounds (GA eq.) /Kg BW through dietary supplementing of microcapsules significantly (*p* < 0.05) improved the average daily gain with respective values of 134.2 and 162.1 mg. Besides, the daily feed intake data was in harmony with bodyweight alterations. The mice challenging with enteropathogenic *E. coli* significantly (*p* < 0.05) reduced the mice's appetite. The inclusion of microcapsules containing phenolic compounds improved the feed intake in a dose-dependent manner similar to the tetracycline as a reference antibiotic.

**Table 5:** The averages of mice body weight changes and feed intake during experiment receiving different treatments^1^

Average	T1	T2	T3	T4	T5	SEM
Average daily weight gain (mg)	155.9^b^	126.3^e^	134.2^d^	162.1^a^	144.3^c^	4.13
Daily feed intake (mg)	2790.6^b^	2604.7^e^	2654.2^d^	2766.8^a^	2642.3^c^	7.39

Different letters in the same raw indicated significant difference (*p* < 0.05)

The analysis was performed in triplicates (*n* = 3)

^1^*T1* Normal diet, *T2* Normal diet + *E. coil* infection, *T3* Normal diet + *E. coil* infection + supplemented with microcapsules (150 mg total phenolic compounds (GA eq.) /Kg BW, T4: Normal diet + *E. coil* infection + supplemented with microcapsules (300 mg total phenolic compounds (GA eq.) /Kg BW, T5: Normal diet + *E. coil* infection + supplemented with oxytetracycline 5 mg /Kg BW

### Liver enzymes and lipid peroxidation status

The obtained results of liver enzymes (ALP, ALT, AST) together with lipid peroxidation have been summarized in Table 6. The results illustrated that the mice challenged by *E. coli* infection (T2) have significantly (*p* < 0.05) elevated the values of these parameters. Meanwhile, the groups receiving the different concentrations of microcapsules (T3 and T4) showed lower levels of liver enzymes and lipid peroxidation values significantly (*p* < 0.05).

**Table 6:** The liver enzymes and lipid peroxidation status of the mice receiving different treatments^1^

	**ALP(U/L)**	**ALT(U/L)**	**AST(U/L)**	**MDA (%)**
T1	148.7^b^	111.9^e^	97.5^e^	100^e^
T2	109.5^e^	231.1^a^	146.3^a^	167^a^
T3	121.8^d^	189.2^b^	129.8^b^	145^b^
T4	154.5^a^	136.5^d^	108.5^d^	122^d^
T5	137.9^c^	149.5^c^	118.3^c^	134^c^
SEM	5.68	4.83	6.79	7.68

Different letters in the same column indicated a significant difference (*p* < 0.05)

The analysis was performed in triplicates (*n* = 3)

^1^T1: Normal diet, T2: Normal diet + *E. coil* infection, T3: Normal diet + *E. coil* infection + supplemented with microcapsules (150 mg total phenolic compounds (GA eq.) /Kg BW, T4: Normal diet + *E. coil* infection + supplemented with microcapsules (300 mg total phenolic compounds (GA eq.) /Kg BW, T5: Normal diet + *E. coil* infection + supplemented with oxytetracycline 5 mg /Kg BW

From this result, it is postulated that the infecting mice with *E. coli* reduced the appetite and induced weight loss due to gastroenteritis. The *E. coli* induced gastroenteritis through adherence to the intestinal mucosa and initiating the production of enterotoxin and cytotoxins [52]. The enterotoxins and cytotoxins reduced the appetite and increased the liver enzymes and lipid peroxidation, resulting in systemic inflammation and oxidative stress induction. The phytobiotic used in this study could improve the weight gain and alleviate the liver enzymes production and lipid peroxidation in the mice challenged by *E. coli* infection. This observation is most probably attributed to the presence of antimicrobial, antioxidant, and anti-inflammatory phenolic compounds (gallic acid, syringic acid, ferulic acid, catechin, ellagic acid, naringin, and chrysin) present in the phytobiotic [53]. These results were consistent with earlier reports indicating the potential of phenolic compounds in reducing the side effects of photogenic bacterial infection in humans and animals [54–56].

### Ileum morphometric analysis

The results of the morphometric analysis of ileum including villus height, villus width, crypt depth, and the number of the goblet cell are presented in Table 7. The overall results demonstrated that the mice group infected with *E. coli* (T2) showed a reduction in the villus height, villus width, and increase in the crypt depth as compared to the uninfected group (T1). The number of goblet cells as a part of the immune system increased significantly (*p* < 0.05) to confront the enteropathogens. Besides, dietary inclusion of microcapsules (150 and 300 mg phenolic compounds /Kg BW) containing phenolic compounds obtained from *P*. *bistorta* root improved the villus height and width together with the reduction in the crypt depth. The microcapsules containing phenolic compounds behaved similarly to the oxytetracycline as reference antibiotics. These results were in agreement with the results of Awad et al. [57] and Fasina et al. [58] who reported the impair in the villus height, villus width, and crypt depth upon the challenge of broiler chicken with enterophategenic bacteria including *C. jejuni* or *Salmonella typhimurium* infection, respectively*.* Further, another study conducted by Mohiti-Asli and Ghanaatparast-Rashti [59] revealed that the inclusion of phenolic compounds in the diet of broiler chicken challenged by *E. coli* improved the morphometric parameters of the small intestine and reduced the presence of *E. coli*.

**Table 7:** Morphometric analysis of ileum in mice received different treatments^1^

	**Villus Height (µm)**	**Villus Width (µm)**	**Crypt Depth (µm)**	Mean Number of Goblet Cells^*^
T1	433^a^	112^b^	124^c^	1.9^d^
T2	258^e^	86^d^	151^a^	3.2^b^
T3	297^c^	98^c^	147^ab^	3.3^ab^
T4	335^b^	126^a^	133^b^	3.6^a^
T5	277^d^	117^ab^	122^c^	2.6^c^
SEM	6.88	5.78	7.31	0.08

Different letters in the same column indicated a significant difference (*p *< 0.05)

The analysis was performed in triplicates (*n* = 3)

^*^(n/100 µm villus height)

^1^T1: Normal diet, T2: Normal diet + *E. coil* infection, T3: Normal diet + *E. coil* infection + supplemented with microcapsules (150 mg total phenolic compounds (GA eq.) /Kg BW, T4: Normal diet + *E. coil* infection + supplemented with microcapsules (300 mg total phenolic compounds (GA eq.) /Kg BW, T5: Normal diet + *E. coil* infection + supplemented with oxytetracycline 5 mg /Kg BW

### Gene expression analysis

The Claudin-1 and Occludin genes are major constituents of the tight junction complexes in the ileum that regulate the permeability of epithelia. Moreover, The Mucin-2 is a gene responsible for the production of related-mucin proteins, polymerizes into a gel of which 80% (w/w) by weight is oligosaccharide side-chains that are insoluble mucus barrier that serves to protect the ileum epithelium against enteropathogens. The expression of these main marker genes involved in permeability and protection showed that enteropathogenic *E. coli* suppressed the expression of claudin-1, claudin-2, occludin, and mucin-2 genes significantly (*p* < 0.05). On the other hand, dietary inclusion of phenolic compounds in the form of microcapsules significantly (*p* < 0.05) up-regulated the expression of these genes (Table 8). These results revealed that infection with *E. coli* interrupted the absorption of nutrients through ileum and reduced the mucin production as a defense mechanism in the ileum against enteropathogenic *E. coli*. These results augur well with the results of average daily weight gain.

**Table 8:** The changes in the expression of tight junction genes in the ileum tissue of mice received different treatments^1^

	T1	T2	T3	T4	T5	SEM
Claudin-1	1.0^c^	0.13^d^	1.14^c^	1.79^a^	1.36^b^	0.14
Claudin-2	1.0^d^	0.21^e^	1.38^c^	2.16^a^	1.47^b^	0.10
Occludin	1.0^d^	0.23^e^	1.62^b^	2.48^a^	1.48^c^	0.09
Mucin-2	1.0^c^	0.42^d^	1.06^c^	2.09^a^	1.64^b^	0.08

Different letters in the same row indicated a significant difference (*p* < 0.05)

The analysis was performed in triplicates (*n* = 3)

^1^T1: Normal diet, T2: Normal diet + *E. coil* infection, T3: Normal diet + *E. coil* infection + supplemented with microcapsules (150 mg total phenolic compounds (GA eq.) /Kg BW, T4: Normal diet + *E. coil* infection + supplemented with microcapsules (300 mg total phenolic compounds (GA eq.) /Kg BW, T5: Normal diet + *E. coil* infection + supplemented with oxytetracycline 5 mg /Kg BW

### E. coli population analysis

**Fig. 3:**
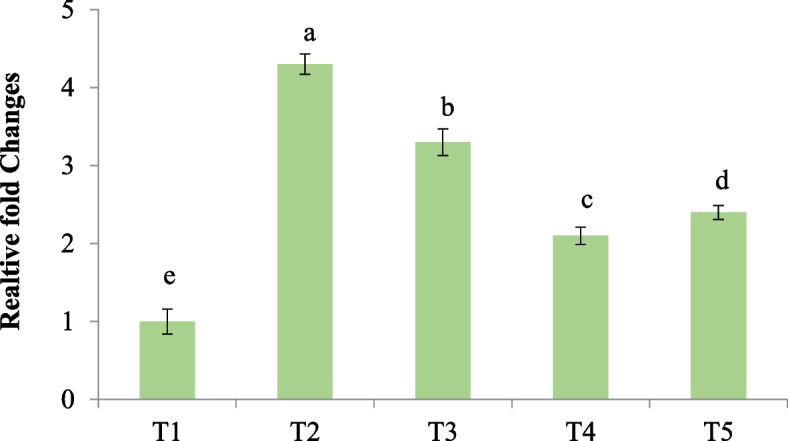
Relative fold changes in the ileum population of *E. coli*. T1: Normal diet, T2: Normal diet + *E. coil* infection, T3: Normal diet + *E. coil* infection + supplemented with microcapsules (150 mg phenolic compounds (GA eq.) /Kg BW, T4: Normal diet + *E. coil* infection + supplemented with microcapsules (300 mg phenolic compounds (GA eq.) /Kg BW, T5: Normal diet + *E. coil* infection + supplemented with oxytetracycline 5 mg /Kg BW. Different letters above charts indicate statistically significant differences among groups (*P* < 0.05). The analysis was performed in triplicates (*n *= 3)

As shown in Fig. 3, the highest *E. coli* population was found in the ileum digesta of the mice challenged by *E. coli*. The dietary addition of microcapsules containing phenolic compounds of *P*. *bistorta* root significantly (*p* < 0.05) reduced the *E. coli* population in the ileum digesta (Fig. 3). The microcapsules containing phenolic compounds behaved similar to the oxytetracycline in reducing the *E. coli* population in the ileum digesta devoid of their differences in the applied concentrations. Hence the microcapsules containing phenolic compounds obtained from *P*. *bistorta* root could play a role as a natural phytobiotic. The release of bioactive phenolic compounds in the ileum section could appear either through the diffusion process or intestinal degradation of wall materials used for the synthesis of microcapsules.

## Conclusion

In this experiment, the phenolic rich extract (PRE) from the root of *Polygonum bistorta* encapsulated in the protecting wall materials (10% WPC, 20% MS, and 70% MD) to form PRE-loaded spherical microcapsules with the mean diameter of 330 nm, PDI of 0.21 and relatively high entrapment efficiency (87.2% w/w). The synthesized microcapsules contained total phenolics of 172.6 ± 9.24 mg GA eq./g dry microcapsules. The microcapsules contained gallic acid, syringic acid, ferulic acid, catechin, ellagic acid, naringin, and chrysin. The in vitro evaluation revealed the antioxidant and antibacterial activity of microcapsules. The dietary supplementation of microcapsules at the concentrations of 150 and 300 mg total phenolic GA eq./Kg BW/day improved weight gain, liver enzymes, lipid peroxidation, gene expression, morphometric characteristics of the ileum and decreased the population of *E. coli* present in the ileum significantly (*p* < 0.05). Consequently, the synthesized microcapsules appeared to be a promising natural antibiotic-alternative called phytobiotic against *E. coli* infection in mice.

## Data Availability

The datasets applied during the current study are available from the corresponding author on reasonable request.

## Abbreviations

ZnO-NPs: Zinc oxide nanoparticle
F.B.S: Fasting Blood Sugar
A.S.T: Aspartate aminotransferase
A.L.T: Alanine aminotransferase
TNF-α: Tumour necrosis factor-alpha
iNOS: Inducible nitric oxide synthase
C.A.T: Catalase
M.D.A: Malondialdehyde
